# Exacerbated Leishmaniasis Caused by a Viral Endosymbiont can be Prevented by Immunization with Its Viral Capsid

**DOI:** 10.1371/journal.pntd.0005240

**Published:** 2017-01-18

**Authors:** Patrik Castiglioni, Mary-Anne Hartley, Matteo Rossi, Florence Prevel, Chantal Desponds, Daniel T. Utzschneider, Remzi-Onur Eren, Haroun Zangger, Livia Brunner, Nicolas Collin, Dietmar Zehn, F. Matthew Kuhlmann, Stephen M. Beverley, Nicolas Fasel, Catherine Ronet

**Affiliations:** 1 Department of Biochemistry, University of Lausanne, Epalinges, Switzerland; 2 Swiss Vaccine Research Institute, Epalinges, Switzerland; 3 Division of Immunology and Allergy, Department of Medicine, Lausanne University Hospital, Lausanne, Switzerland; 4 Department of Molecular Microbiology, Washington University School of Medicine, Saint Louis, Missouri, United States of America; 5 Department of Medicine, Division of Infectious Disease, Washington University School of Medicine, Saint Louis, Missouri, United States of America; Universidade Federal de Minas Gerais, BRAZIL

## Abstract

Recent studies have shown that a cytoplasmic virus called *Leishmaniavirus* (LRV) is present in some *Leishmania* species and acts as a potent innate immunogen, aggravating lesional inflammation and development in mice. In humans, the presence of LRV in *Leishmania guyanensis* and in *L*. *braziliensis* was significantly correlated with poor treatment response and symptomatic relapse. So far, no clinical effort has used LRV for prophylactic purposes. In this context, we designed an original vaccine strategy that targeted LRV nested in *Leishmania* parasites to prevent virus-related complications. To this end, C57BL/6 mice were immunized with a recombinant LRV1 *Leishmania guyanensis* viral capsid polypeptide formulated with a T helper 1-polarizing adjuvant. LRV1-vaccinated mice had significant reduction in lesion size and parasite load when subsequently challenged with LRV1+ *Leishmania guyanensis* parasites. The protection conferred by this immunization could be reproduced in naïve mice via T-cell transfer from vaccinated mice but not by serum transfer. The induction of LRV1 specific T cells secreting IFN-γ was confirmed in vaccinated mice and provided strong evidence that LRV1-specific protection arose via a cell mediated immune response against the LRV1 capsid. Our studies suggest that immunization with LRV1 capsid could be of a preventive benefit in mitigating the elevated pathology associated with LRV1 bearing *Leishmania* infections and possibly avoiding symptomatic relapses after an initial treatment. This novel anti-endosymbiotic vaccine strategy could be exploited to control other infectious diseases, as similar viral infections are largely prevalent across pathogenic pathogens and could consequently open new vaccine opportunities.

## Author Summary

About 80% of leishmaniasis infections result in cutaneous manifestations with a broad symptomatic spectrum, ranging from self-healing localized to disseminated lesions. The mechanism behind these latter aggravated forms of leishmaniasis is still poorly understood. One possible factor is an endosymbiotic RNA virus identified in the cytoplasm of several *Leishmania* species (*Leishmania* RNA virus 1). LRV1 acts as a virulence factor, causing a destructive hyper-inflammatory response. In this study, we tested the prophylactic potential of a vaccine formulated with a recombinant LRV1 capsid and a T helper 1-polarizing adjuvant. Our approach conferred significant protection against LRV1+ *Leishmania guyanensis* infection, decreasing lesional inflammation and parasite burden. Further analysis demonstrated that this vaccine induced a potent T helper 1 response. Consequently, we propose that the LRV1-capsid is a promising vaccine component in order to reduce clinical complications (e.g. symptomatic relapses) in areas endemic to LRV1 co-infected *Leishmania* species. Taken together, we present an original strategy, whereby targeting the pervasive intracellular viruses within pathogens may reduce pathologic inflammation and offer an extra-genetic candidate that may circumvent escape mutations or poor response to drug treatment.

## Introduction

*Leishmania* are protozoan parasites belonging to the family *Trypanosomatidae* of the Order *Kinetoplastida*, and are insect-vectored human parasites distinguished as single-celled flagellate organisms with a digenetic lifecycle. These obligate intracellular parasites are capable of infecting various mammals, including humans, where they cause the devastating and neglected disease, leishmaniasis [1–3]. There are different clinical manifestations of leishmaniasis: cutaneous (CL), visceral (VL) and mucocutaneous leishmaniasis (MCL). In this latter case, the cutaneous form may present with a range of complications, that is generally characterized by intensely inflamed and chronic lesions, which metastasize to secondary sites and are refractory to current therapies [4,5]. These symptomatic differences are thought to be principally determined by the phylogeny of the infecting parasite [6]. The *Leishmania* genus is divided in two major subgenera: *Leishmania* (*L*. *Leishmania*) and *Leishmania Viannia* (*L*. *Viannia*). Clinical complications of hyper-inflammation and infectious metastasis are mainly found within the *L*. *Viannia* subgenus, which includes *L*. *guyanensis* (*Lg*), *L*. *braziliensis* (*Lb*) and *L*. *panamensis* (*Lp*) [7–9]. However, mucocutaneous and diffuse forms of infection have also been attributed to *L*. *Leishmania aethiopica* (*Lae*) [10–12]. The exact factors implicated in these types of metastatic leishmaniasis are poorly understood but could be attributed to either host genetic polymorphisms or species-specific parasite virulence factors [13]. A recently proposed parasitic metastatic factor is a cytoplasmic virus within certain *Leishmania* parasites and called *Leishmaniavirus* 1 (LRV1). It was shown that the potent anti-viral immune response against this endosymbiont could explain the exaggerated inflammation seen in LRV1-carrying strains of *Lg* [7]. The host response is carried out via the innate dsRNA receptor, Toll-like receptor 3 (TLR3), which triggers anti-viral immune response mediated by type-I interferons (IFNs), interleukin (IL)-6 and tumour necrosis factor (TNF)-α. Together, these factors were shown to increase parasite survival and lesional swelling in a murine model of disease [7]. Since then, LRV1 has also been linked to virulence in humans, where patients infected by LRV1-carrying *Lg* and *Lb* parasites have higher rates of treatment failure and disease relapse [4,5] and is sometimes associated with increased occurrence of mucocutaneous leishmaniasis [14]. The presence of this endosymbiotic virus was first described several decades ago, when virus-like-particles were found in the cytoplasm of *Lg* strain MHOM/SR/81/CUMC1A [15] and confirmed in the *Lg* M4147 strain [16]. Viral particles are observed as 30-50nm icosahedral particles within parasite cytoplasm and are composed of a dsRNA genome associated to an RNA-dependent RNA polymerase (RDRP) and an 83kDa capsid protein. Genetic analysis has classed LRV1 in the *Totiviridae* family [17], a group of RNA viruses infecting a large spectrum of organisms such as protozoa, yeast, fungi, plants, arthropods, shrimps and also some vertebrates [18–23]. Several *Leishmania* species are known to carry LRVs. LRV is named LRV1 in species of the *L*. (*Viannia*) subgenus and LRV2 in *L*. (*Leishmania*) *aethiopica* [24], and in *L*. *major* [25]. These parasite species contribute to a significant proportion of the burden of leishmaniasis and its clinical complications [4,5,14] making an LRV-targeted therapeutic or prophylactic intervention particularly promising.

As the pathogenesis of LRV1 is mediated by its ability to solicit potent inflammation via TLR3 activation [7], we tested whether immunization with an LRV1 capsid polypeptide could target infected cells and control parasite load by launching an early cell-mediated response and thus limit the LRV-mediated pathology. In this report, we demonstrated the ability of a vaccine formulated with recombinant LRV1 capsid to generate protective immunity against LRV1+ *Lg* -mediated disease.

## Materials and Methods

### Ethics statement

All animal protocols in this publication were approved by the Swiss Federal Veterinary Office (SFVO), under the authorization numbers 2113.1 and 2113.2. Animal handling and experimental procedures were undertaken with strict adherence to ethical guidelines set out by the SFVO and under inspection by the Department of Security and Environment of the State of Vaud, Switzerland.

### Mice

C57BL/6 mice (5 to 6 weeks old) were purchased from Harlan Laboratories (Netherlands). The entire study was performed using C57BL/6 wild type mice.

C57BL/6 IFN-γ-/- mice were purchase from Jackson laboratory.

Mice were maintained under pathogen-free conditions at the animal facility of the Centre of Immunity and Immunology, Lausanne (Switzerland).

### Recombinant protein production and protein quantification and analysis

M5313 LRV1 and M41447 LRV1 capsids sequences used in this study have been identified in WHI/BR/78/M5313 and MHOM/BR/75/M4147 Brazilian *L*. *guyanensis* strains respectively. They show 99.46% identity. The LRV1 capsid open reading frame (Genbank accession number: JX313126) was cloned in frame into a pET-28a *E*. *coli* expression vector (Merck) with a His-tag sequence placed at the C-terminus and was amplified from a cDNA preparation of *Leishmania guyanensis* M5313 parasites [26]. Colonies were taken and grown in Magic Media (*E*.*coli* expression Medium, Invitrogen). Pellet was then resuspended in a lysis buffer (50 mM NaH_2_PO_4_, 300 mM NaCl and 10 mM Imidazole and H_2_O to reach a total volume of 50 mL), then was lysed again in the same buffer with the addition of 2% SDS (20%). After a dialysis step to remove SDS, the lysate was passed through a Nickel column.

Purified recombinant protein was quantified by spectral quantification using a Nanodrop spectrophotometer (Thermo scientific). A total of 0.1 μg of this preparation was separated in a 10% polyacrylamide denaturing gel, then stained with Coomassie brilliant blue G250 (Fluka) (0.05% G250, 10% acetic acid, 30% ethanol) and washed with distaining solution (10% acetic acid, 30% ethanol, 60% water) O/N to verify its quality (S1 Fig, left gel).

Quantified LRV1c protein (0.1μg) was also separated in a 10% polyacrylamide denaturing gel, and transferred onto a nitrocellulose membrane over an electric gradient. Transferred LRV1c protein was visible by Ponceau Red staining. After a 1h blocking step in 5% powdered milk diluted in TBS + 0.05% Tween20 (TBS-Tw), the membrane was incubated overnight at 4°C with the g018d53 anti-capsid polyclonal antibody (1:5000 in 1% milk and TBS-Tw) [26]. Following 4 washes in TBS-Tw, 15 min at RT, the membrane was incubated for 1 h with an anti-rabbit IgG antibody coupled to peroxidase (Promega) (1:2500 in 1% milk TBS-Tw), washed again 4x in TBS-Tw and finally revealed by ECL chemiluminescence (Amersham) (S1 Fig, right gel).

### Parasites and cell culture

Previously we described a clonal derivative of the LRV1+ strain of *L*. *guyanensis* M4147 (MHOM/BR/75/M4147), containing a firefly luciferase (ffLUC) gene integrated stably into the small subunit gene of the ribosomal RNA locus (LgM4147/SSU:IR2SAT-LUCb LRV1+) [27]. In work to be described elsewhere, clonal lines retaining or lacking LRV1 were obtained following brief drug treatments [28], resulting in isogenic LRV1+ or LRV1- lines. These lines fully recapitulate the LRV1+ versus LRV1- phenotypes found in prior studies [7] using isogenic lines developed by Ro and Patterson after transfection, drug treatment and prolonged culture *in vitro* (Kuhlman and Beverley, in preparation). Parasites were cultured *in vitro* as promastigotes at 26°C in freshly prepared Schneider’s insect medium (Sigma) supplemented with 10% heat-inactivated fetal bovine serum (PAA), 10mM HEPES and 50U/mL penicillin/streptomycin (Animed), 0.6mg/L biopterin and 5mg/L hemin (Sigma-Aldrich). Each passage yielded infectious metacyclic promastigotes after 6 days and cultures were kept no longer than 5–6 passages.

### Mouse infection

Mice were injected sub-cutaneously with 3 x 10^6^ parasites of LRV1+ *Lg* or LRV1- *Lg* into the hind footpads. Change in footpad swelling was measured weekly using a Vernier caliper as a proxy for disease progression.

### Antibody response

Blood samples were collected from LRV1+ *Lg* or LRV1- *Lg* infected mice 8 weeks after infection. Once coagulated, samples were centrifuged at maximum speed and serum was collected and kept at -20°C until analysis. ELISA against LRV1c was performed on the serum to quantify the level of specific IgGs against the viral capsid. Briefly, ELISA plates (Maxisorp, Nunc) were coated with 5μg/mL LRV1c in PBS O/N at 4°C. The plate was then washed with PBS-Tween (0.05% Tween) and blocked with 1x assay diluent (eBioscience) for 1 hour at RT.

Sera from LRV1+ *Lg*, LRV1- *Lg* or uninfected control mice were then plated in a 3 fold serial dilution with a starting concentration of 1:50. After 2h of incubation and washes with PBS- Tween, a secondary antibody against total IgGs (Jackson Immunoresearch, goat anti-mouse, 1:5000), IgG2c (rat anti-mouse, 1:3000, BD bioscience) or IgG1 (rat anti-mouse, 1:5000, BD bioscience) coupled with Biotin was added to the plate and incubated for 1 hour at RT before adding during 20 min streptavidin-HRP (1:250, eBioscience). Quantification was determined by colorimetric assay using TMB substrate according to supplier’s instructions (eBioscience).

### Adjuvants

Squalene-in-water emulsion (SWE) was prepared as previously described and contained a metabolizable oil (squalene 3.9%, w/v), sorbitan trioleate (0.47%, w/v), and polyoxyethylene (80) sorbitan monooleate (0.47%, w/v) dispersed in 10 mM citrate buffer at pH 6.5 [29]. Liposome containing QS-21 saponin (LQ) were manufactured by a lipid film-rehydration method followed by extrusion and contained 2.5 mg/mL of cholesterol and 10 mg/mL of DOPC (1, 2-dioleoyl-sn-glycero-3-phosphocholine) in PBS at pH 7.2. The saponin QS-21 adjuvant was prepared as 1 mg/mL solution in DPBS (Dulbecco’s phosphate buffer saline, without Ca or Mg). The adjuvant LQ was obtained by mixing the liposome suspension and the QS-21 solution 1:1 v/v. SWE containing Monophosphoryl lipid A (SM) was prepared as follows MPL (Monophosphoryl Lipid A, from Salmonella enterica serotype Minnesota Re 595, Sigma-Aldrich) was suspended in WFI (0.2% DMSO (Applichem) in sterile water) at 1 mg/mL, ultrasonicated with a probe sonicator and sterile filtered (0.20 μm). This solution was added to the SWE emulsion (1:2.5 v/v). All the above adjuvants were prepared by the Vaccine Formulation Laboratory (VFL), University of Lausanne (Switzerland). Montanide ISA 720 was purchased from Seppic and CpG ODN-1826 from InvivoGen and Trilink.

### Preparation of vaccine formulations

*LRV1c + SFI*: 168 μL of 1.2 mg/mL LRV1c protein solution in PBS (Sigma) (202 μg of protein) were added to 832 μL of SFI (0.2% DMSO (Applichem) in sterile 0.9% NaCl (SFI, Braun).

*LRV1c + SWE*: 168 μL of 1.2 mg/mL LRV1c protein solution in PBS were diluted into 232 μL of WFI. The solution was added to 500 μL of SWE. The mixture was vortexed 5s and left standing at least 30 minutes at room temperature before administration.

*LRV1c + SM*: 168 μL of 1.2 mg/mL LRV1c protein solution in PBS were diluted into 216 μL of WFI and 300 μL of the solution were added to 700 μL of SM. The mixture was vortexed 5s and left standing at least 30 minutes at room temperature before administration.

*LRV1c + LQ*: 168 μL of 1.2 mg/mL LRV1c protein solution in PBS were diluted into 432 μL of sterile PBS. 600 μL of the solution were added to 400 μL of LQ and left standing at least 30 minutes at room temperature before administration.

*LRV1c + Montanide ISA 720*: 700 μL of Montanide ISA-720 were added to 168 μL of 1.2 mg/mL of LRV1c protein solution in PBS by using two syringes connected by a female luer lock adapter until a homogenous emulsion was obtained (50 times).

*LRV1c + CpG*: 168 μL of 1.2 mg/mL LRV1c protein solution in PBS were diluted into 782 μL in PBS (Sigma) and added 50 μL (20mg/mL) of CpG ODN-1826 (InvivoGen), for a final amount of 10 μg of LRV1c and 50 μg of CpG ODN-1826 for each mouse.

### Serum transfer

Sera was collected as described above and re-injected intra-peritoneally (IP). Each mouse was challenged twice with 200 μL, once 2 days before infection, and a second time 2 days after infection. The presence of LRV1c-antibodies was assessed by an ELISA assay as described above, to confirm the efficacy of the transfer (S4 Fig).

### Mouse immunization

Age-matched (4–5 week old) female mice were inoculated intramuscularly (IM) (50μl) with 10μg of LRV1 capsid recombinant protein in PBS formulated with the various adjuvant emulsions as described above. Mice were immunized twice, fifteen days apart, and infected 21 days after the second immunization. Following immunization with CpG-ODN 1826 formulation, mice were challenged with 10μg of LRV1 capsid supplemented with 50μg of CpG and then completed with PBS (endotoxin-free, Sigma). Three immunizations 15 days apart were performed before infection. The last immunization was performed 21 days before infection.

### Parasite quantification

At the peak of infection (week 4), parasite burden was quantified using an *in vivo* imaging system to detect luminescence emitted by luciferase-transfected parasites. Mice were injected intra-peritoneally with D-Luciferin sodium salt (Regis technologies) prepared in PBS at a final concentration of 150 mg/kg. After 7 min incubation, mice were anesthetized by continuous gas anaesthesia (isoflurane) during 3 min and imaged using a Xenogen Lumina II imaging system (IVIS, 10 min exposure time). Total photon flux over footpad lesions was assessed using the associated software (Living Image).

### Cytokine analysis at the peak of infection by ELISA

Draining popliteal lymph nodes were isolated and cultured at a concentration of 1x10^6^cells/mL in DMEM (Gibco) supplemented with 10% heat inactivated FBS, 1% penicillin/streptomycin, 1% Hepes (Sigma-Aldrich) (complete DMEM). Cells were then stimulated with UV treated (3 minutes) 2x10^5^/mL *L*. *guyanensis* promastigotes for 72 hours at 37°C and 5% CO_2_. After 72h incubation supernatants were collected and analyzed in duplicate by ELISA. IL-10, IL-4, IFN-γ (eBioscience) were quantified according to the manufacturer’s instructions and read on a Synergy^™^ HT Multi-Mode Plate Reader (Biotek Instruments, Switzerland).

### Cytokine analysis in vaccinated mice

Bone marrow macrophages cells (BMMs) were prepared using bone marrow cells collected from 4–5 week old C57BL/6 mice. Cells were cultured in 10mL complete DMEM supplemented with 50ng/mL M-CSF like described in [30]. Bone marrow cells were cultured 3 days at 37°C and 5% CO_2_. At day 3, 5mL of complete DMEM supplemented with 50ng/mL were added to the culture. After 6 day, BMMs were plated in a 48 well plate and infected with LRV1+ *Lg* or LRV1- *Lg* (MOI 5:1) and 2 μg/mL of LRV1c for 20 hours, maintained at 37°C in 5% CO_2_. Macrophages were then fixed 10 minutes with 4% PFA. Spleen cells from vaccinated or control mice (7 days after the third inoculation) were added to previously infected BMMs with or without BrefeldinA in order to analyses cytokines by intracellular FACS or by ELISA. After 4 hours of incubation, spleen cells incubated with BrefeldinA (5μg/mL) were stained extracellularly with anti FcR antibodies (24G2, ebioscience) and then with α-CD4-BrilliantViolet421 (1:500, clone M1/70, Biolegend) α-CD8-PerCP (1:500, clone 53–6.7, Biolegend) and α-CD3-FITC (1:1000, clone 145-2C11, eBioscience) and then fixed 30 min RT with a fixation Buffer according to manufacturer’s instructions (eBioscience). Cells were then permeabilized with Perm Buffer (eBioscience) and stained intracellularly with α-IFN-γ-PE (1:100, clone XMG1.2, Biolegend). Cells were then analyzed by FACS using LSRII BD. Spleen cell supernatants cultured without BrefeldinA were then analyzed 72 hours post incubation by ELISA to detect IL-10, IL-4 and IFN-γ (eBioscience) as described previously.

### T cell purification and transfer

One week after the third recall, lymphocytes were collected from spleen of immunized or control group mice. Pelleted cells were resuspended in RobosepTM buffer (Stemcell technologies) at a final concentration of 10^8^ cells/mL. 5*10^8^ cells per group were purified by CD3+, CD8+ or CD4+ negative selection using an EasySep isolation kit (Stemcell Technologies). Cells were then injected intravenously (IV) in 4–5 week old C57BL/6 mice (10^7^ cell in 300μl). The remaining cells were marked with α-CD3-FITC (1:1000, clone 145-2C11, eBioscience), α-CD4-BrilliantViolet421 (1:500, clone M1/70, Biolegend) or α-CD8-PerCP (1:500, clone 53–6.7, Biolegend) stain and analyzed by FACS to assess the purity of the cell suspension, purity was always superior to 95%.

## Results

### LRV1 capsid is immunogenic

In order to test whether the LRV1 capsid is recognized by the murine immune system and to assess its immunogenicity, C57BL/6 mice were infected with isogenic *Lg* parasites either, with or without, LRV1 (hereafter named: LRV1+ *Lg* or LRV1- *Lg* respectively).

**Fig 1:**
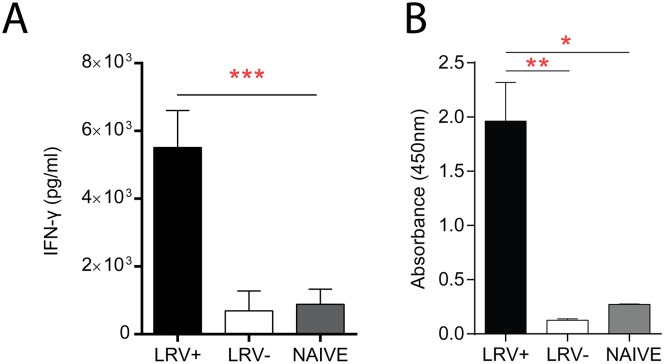
LRV1 capsid is immunogenic. C57BL/6 mice infected with LRV1+ *Lg* or LRV1- *Lg* parasites. (A) Mice were sacrificed 4 weeks post infection and IFN-γ levels were analysed in the supernatant (SN) of restimulated cells with recombinant LRVc by ELISA to assess the cellular immune response against LRVc (white LRV1- *Lg* and black LRV1+ *Lg*) or control mice (grey). (B) Mice were bled 8 weeks post infection. Sera were collected and tested for the presence of IgG specific to the LRV1c protein by ELISA (see Materials and Methods). IgG were analyzed form sera of infected mice (white LRV1- *Lg* and black LRV1+ *Lg*) or control mice (grey). Results are means±SEM. Statistical significance tested by a two-way ANOVA, using Prism5 Graphpad software (n = 5, *: P<0.05, **: P<.005, ***: P<0.0005). Representative of 2 independent experiments.

The cellular immune response was analysed 4 weeks post infection. Draining lymph nodes (dLN) cells were collected and restimulated for 3 days using a purified *E*. *coli* recombinant viral capsid expressed as a His-tagged polypeptide [26]. Results showed an intense production of IFN-γ in mice previously infected with LRV1+ *Lg* and almost no response in mice infected with LRV1- *Lg* (Fig 1A). In addition, to analyse the humoral immune response against LRV1, blood was recovered approximately 8 weeks post infection, at the time of lesion resolution to quantify specific anti-LRV1 capsid IgGs by Enzyme-Linked Immunosorbent Assay (ELISA). As shown in Fig 1B, we detected IgGs against the LRV1 capsid in mice infected with LRV1+ *Lg* but not with LRV1- *Lg*, suggesting that the viral capsid is processed and presented by antigen presenting cells and specific anti-capsid antibodies were produced via a B cell response.

### Immunization with recombinant LRV1 capsid alone induces a mixed Th2/Th1-type response

**Fig 2:**
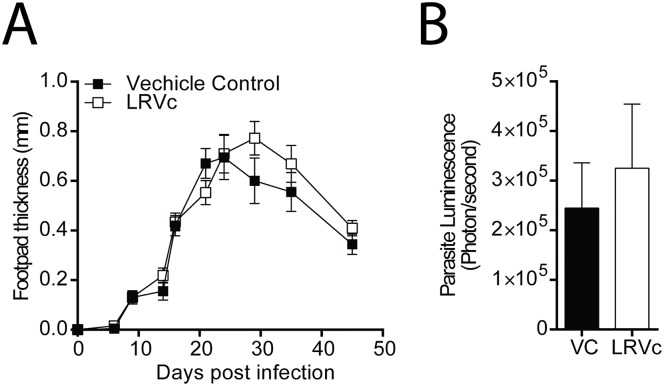
Immunisation with LRV1c alone did not induce protection. Mice were injected twice intramuscularly with 10μg of LRV1c, 3 weeks apart. After the second vaccination, mice were infected in the hind of the footpad with 3x10^6^ LRV1+ *Lg* parasites. Footpad thickness was measured weekly (A) and parasite load (B) was measured by bioluminescence at 4 weeks post-infection. (B) Statistical significance tested by a two-way ANOVA, using Prism5 Graphpad software (n = 5, *: P<0.05, **: P<0.005, ***: P<0.0005). Representative of 5 independent experiments.

To assess the protective capacity of LRV1c, C57BL/6 mice were immunized (IM) with 10 μg of LRV1c. Immunizations were performed twice (fifteen days apart), and mice were infected with LRV1+ *Lg* parasites three weeks after the second immunization. No differences were observed between LRV1c-immunized mice, or the non-immunized control group, in either footpad lesion size or in parasite burden (Fig 2A and 2B). To understand why the immunization failed to control the exacerbation caused by LRV1, we investigated which type of immune response was raised against the capsid. Indeed, the humoral response against LRV1c was a mixed Th1/Th2 response, shown by the presence of high levels of IgG2 and IgG1 in sera of immunized animals (S2 Fig), possibly explaining the lack of protection and demonstrating that specific adjuvants should be included in the vaccine formulation.

### Immunization with adjuvanted LRV1c confers significant protection against LRV1+ *Lg* infection

**Fig 3:**
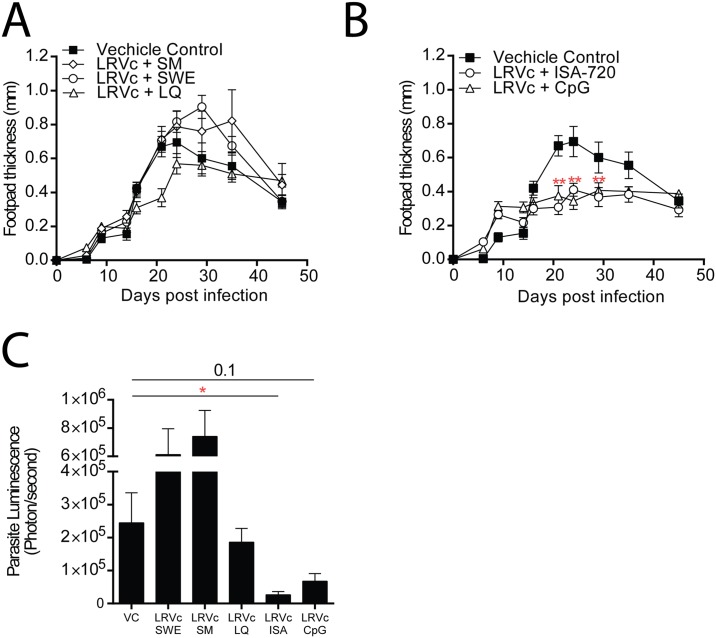
LRV1c formulated with CpG-ODN or ISA-720 induced a strong protection in C57BL/6 mice. LRV1c was formulated with various adjuvants like: squalene-in-water emulsion (SWE), squalene-in-water emulsion with MPL (SM), water-in-oil emulsion (Montanide ISA-720), liposome-saponin-based adjuvant (LQ) and CpG ODN-1826. Mice were vaccinated twice 21 days apart and then infected with LRV1+ L.g parasites 21 days after the second inoculation. (A-B) Footpad lesion size was measured weekly and parasite load was quantified by bioluminescence at the peak of infection (C). Results are means±SEM. (A-B) Statistical significance tested by a two-way ANOVA, using Prism5 Graphpad software (n = 5, *: P<0.05, **: P<.005, ***: P<0.0005). Representative of 2 independent experiments. C) Statistical significance tested by a 2-tailed Student’s t-test using Prism5 Graphpad software (n = 5, *: P<0.05, **: P<0.005, ***: P<0.0005 vs Vehicle Control). Representative of 2 independent experiments.

In a preliminary experiment, several adjuvant systems were tested in LRV1c formulations: a squalene-in-water emulsion (SWE), a squalene-in-water emulsion containing Monophosphoryl lipid A (SM), a water-in-oil emulsion (Montanide ISA-720), liposomes containing QS-21 saponin (LQ) and CpG ODN-1826 (CpG). After two immunizations with these preparations, mice were infected with LRV1+ *Lg* and disease progression was followed by measuring footpad swelling and parasite burden. Results showed that LRV1c formulated with either Montanide ISA-720 or CpG were able to confer significant reduction in footpad lesion size (Fig 3A and 3B), as well as a strong reduction in parasite load at the peak of infection (Fig 3C). However, further analysis showed that ISA-720 induced continuous activation of the immune system caused by the limited reabsorption of the adjuvant and its persistence as a sterile abscess at the site of injection. Therefore, we decided to limit our study to a formulation based on LRV1c (10μg/mouse) containing CpG (50 μg/mouse) (LRV1c+CpG).

### LRV1c vaccination confers protection in an LRV1-specific manner

**Fig 4:**
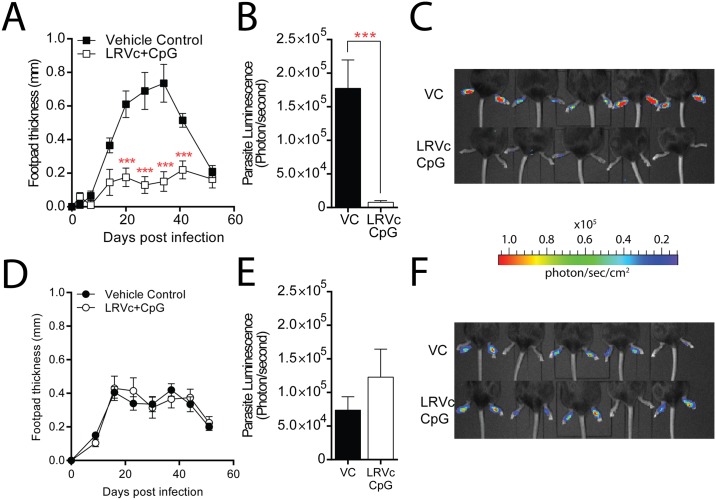
Immunization with LRV1c+CpG was specific to the *Leishmania* RNA virus capsid. C57BL/6 mice were immunized 3 times IM, 15 days apart with LRV1c+CpG or PBS as a control. 21 days after the third vaccination, mice were infected with either LRV1+ *Lg* or LRV1- *Lg*. (A) Change in footpad swelling of mice immunized with LRV1c+CpG (white square) or vehicle control (black square) and infected with LRV1+ *Lg*. (B-C) *In vivo* bioluminescence of mice challenged with LRV1c+CpG (white) or vehicle control (black) and infected with LRV1+ *Lg* at the peak of infection. (D) Change in footpad swelling of mice immunized with LRV1c+CpG (white circle) or vehicle control (black circle) and infected with LRV1- *Lg*. (E-F) *In vivo* bioluminescence of mice immunised with LRV1c+CpG (white) or vehicle control (black) and infected with LRV1- *Lg* at the peak of infection. Results are means±SEM. (A and D) Statistical significance tested by a two-way ANOVA, using Prism5 Graphpad software (n = 5, *: P<0.05, **: P<.005, ***: P<0.0005). (B-E) Statistical significance tested by a 2-tailed Student’s t-test using Prism5 Graphpad software (n = 5, *: P<0.05, **: P<0.005, ***: P<0.0005). Representative of 8 independent experiments.

Having defined a possible adjuvant, we confirmed the protective effect of LRV1c vaccine formulated with CpG (LRV1c+CpG) *in vivo*. C57BL/6 mice were infected with either LRV1+ *Lg* or LRV1- *Lg*, 3 weeks after three immunizations to enhance protection. Significant protection against LRV1 was only conferred by an LRV-specific response, as protection was found in LRV1+ *Lg* infected mice (Fig 4A, 4B and 4C) but not in their LRV1- *Lg* infected counterparts. Indeed, LRV1- *Lg* infected mice showed no difference in disease progression compared to non-immunized mice in terms of footpad swelling and parasite burden at the site of infection (Fig 4D, 4E and 4F). We observed a significant decrease in lesion size and parasite burden after three rounds of immunization compared to the previous experiments, where mice were immunized only twice with LRV1c+CpG (Fig 3B and 3C). In parallel, we also checked that CpG did not induce any protective effect on its own, as it had no effect on disease progression or parasite load when used alone (S3 Fig).

### The humoral immune response does not play a role in the LRV1 protection mediated by LRV1c vaccination

**Fig 5:**
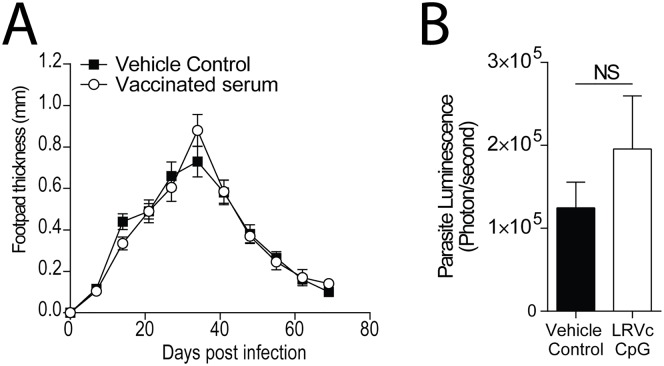
B cells played no role in the protection mechanism. Serum from LRV1c+CpG immunized or non-immunized mice was re-injected in naïve C57BL/6 mice. (A) Change in footpad swelling of mice receiving immunized serum (white square) versus naïve serum (black square). Sera were administrated two days before and after the infection with LRV+ *Lg*. (B) *In vivo* bioluminescence of mice challenged with LRV1c+CpG (white) or non-immunized (black) and infected with LRV1+ *Lg* at the peak of infection. Statistical significance tested by a 2-tailed Student’s t-test using Prism5 Graphpad software (n = 5, *: P<0.05, **: P<0.005, ***: P<0.0005). Representative of 2 independent experiments.

In order to investigate the mechanism of protection against LRV1 induced by LRV1c immunization, we assessed the role of B cells in our murine model. To this end, uninfected C57BL/6 mice previously immunized with LRV1c+CpG were bled and serum was tested in order to see if it could confer protection in non-immunised mice. Thus, by transferring serum, we investigated whether reduction in lesion size and parasite burden was conferred by anti-LRV1c antibodies. Sera from vaccinated or PBS-challenged mice (vehicle control, VC) were re-injected into naïve C57BL/6 mice. Subsequently mice were infected with LRV1+ *Lg*. We confirmed the presence of LRV specific IgGs in recipient mice throughout the infection (S4 Fig). However, no protection could be observed neither in terms of lesion size or parasite load at the site of infection (Fig 5A and 5B). We could conclude that fundamental components of the humoral immune system did not play any relevant role in the protection mechanism conferred by LRV1c+CpG immunization.

### T cells from LRV1c + CpG vaccinated mice respond specifically to LRV1 infection

**Fig 6:**
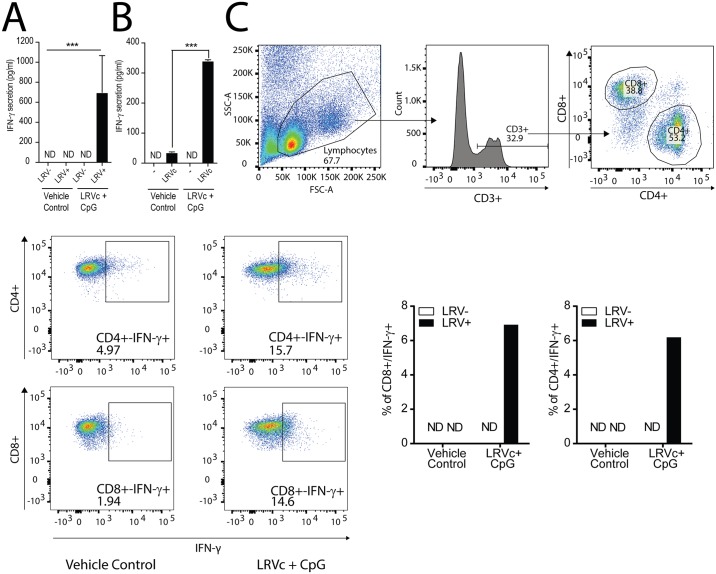
LRV1c formulated with CpG-ODN 1826 induced LRV1c specific IFN-γ secreting T cells. Seven days after the third challenge, LRV1c+CpG, or vehicle control mice were sacrificed and splenocytes were restimulated with BMM infected with LRV1+ *Lg*, LRV1- *Lg* or stimulated with LRV1c (see Material and Method). (A) IFN-γ secretion from vehicle control mice, or LRV1c+CpG incubated with BMM stimulated with LRV1c. (B) IFN-γ secretion from vehicle control mice or LRV1c+CpG incubated with BMM infected with LRV1+ *Lg*, or LRV1- *Lg*. (C) Intracellular FACS on dLN cells stimulated with LRV1+ *Lg* or LRV1- *Lg* infected macrophage in presence of BrefeldinA. Cells were gated firstly on CD3+ T cells, then on CD8+ and CD4+, and finally on IFN-γ secretion. Results are means±SEM. (A-B) Statistical significance tested by a 2-tailed Student’s t-test using Prism5 Graphpad software (n = 3, *: P<0.05, **: P<0.005, ***: P<0.0005 vs Vehicle Control). Representative of 4 independent experiments. (C) Statistical significance tested by a 2-tailed Student’s t-test using Prism5 Graphpad software (n = 1, *: P<0.05, **: P<0.005, ***: P<0.0005 vs Vehicle Control). Representative of 3 independent experiments.

We then investigated the presence of LRV1c-specific T cells in vaccinated mice. Splenocytes were purified from vaccinated and VC mice and re-stimulated *ex vivo* with bone marrow derived macrophages (BMMs) infected with either LRV1+ *Lg* or LRV1- *Lg*, or stimulated with LRV1c. ELISA analysis of the supernatants from LRV1c-immunized mice showed that T cells from these mice produced an efficient cytokine response to macrophages infected by LRV1+ *Lg* parasites but not to those infected by LRV1- *Lg* (Fig 6A). A similar response was also generated when T cells were co-cultured with LRV1c stimulated macrophages (Fig 6B). In contrast, lymphocytes from VC mice, produced a negligible reaction to all the combinations. Subsequently, to identify the cell type responsible for this pronounced cytokine secretion we performed intracellular FACS analysis and found that CD3+ T cells were responsible for the IFN-γ secretion observed in the ELISA experiment. We identified these cells as CD4+ and CD8+ (Fig 6C). These results demonstrated the presence of LRV1c-specific T cells capable of producing IFN-γ in immunized mice with LRV1c+ CpG.

### LRV1c vaccination results in a Th1 immune response

To further characterize the protective immune response, we analysed cytokine production in lymph nodes draining the leishmanial footpad lesions (dLN). In particular, we investigated the major Th1 and Th2 differentiation cytokines IFN-γ and IL-4, as well as IL-10 secretion. To this end, C57BL/6 mice were immunized, or not, with LRV1c as described previously, and after 4 weeks of infection with LRV1+ *Lg*, lymphocytes from dLNs were collected and re-stimulated *ex-vivo* with either LRV1+ *Lg* or LRV1- *Lg* parasites, or recombinant LRVc. Thus, by comparing re-stimulations it was possible to discriminate between the immune response directed toward parasite alone (i.e. LRV1- *Lg* re-stimulation) and the response directed toward the entire LRV1 complex (i.e. the difference between LRV1- *Lg* and LRV1+ *Lg* re-stimulation).

**Fig 7:**
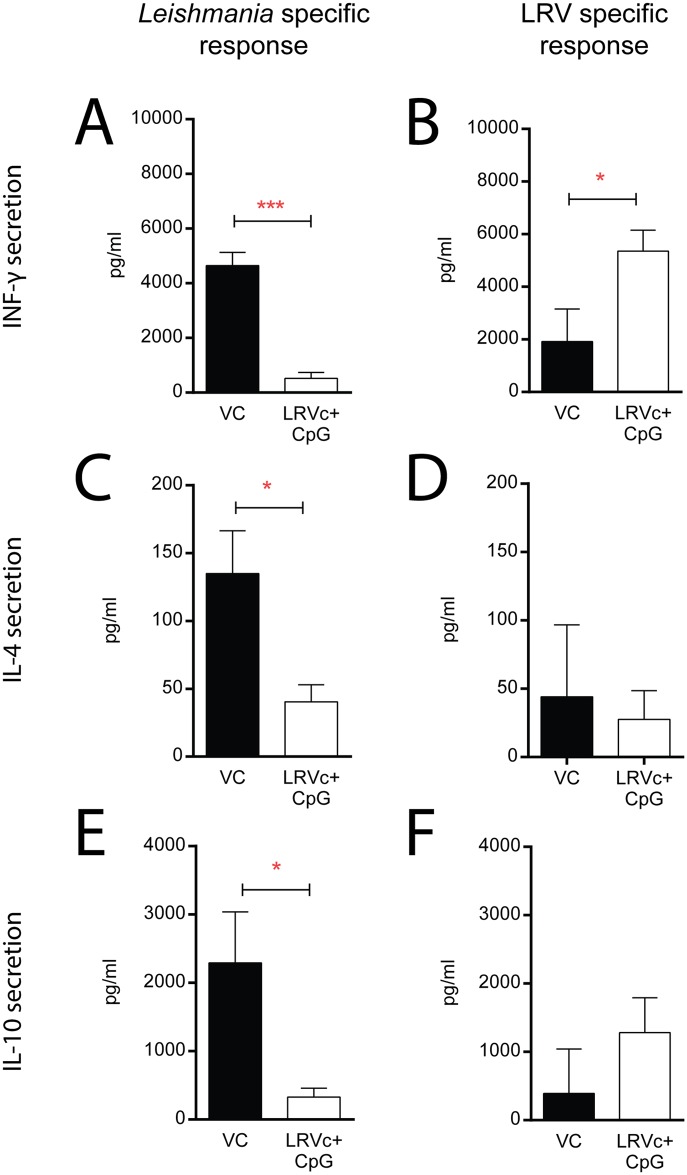
LRV1c formulated with CpG-ODN 1826 induced a strong Th1 immune response against the viral capsid. C57BL/6 mice vaccinated or not with the LRVc+ CpG were infected with LRV1+ *Lg* parasites and at 4 weeks post infection their dLN cells were restimulated ex vivo with either LRV1+ *Lg* (right side) or LRV1- *Lg* parasites (left side). By comparing these two re-stimulations, we discriminated between the immune response directed against the parasite (i.e. LRV1- *Lg* re-stimulation) and the response directed toward the LRV1 capsid (i.e. the difference between LRV1- *Lg* and LRV1+ *Lg* re-stimulation). IFN-γ, IL-4 and IL-10 level were detected by ELISA 72h after re-stimulation. Results are means±SEM. Statistical significance tested by a 2-tailed Student’s t-test using Prism5 Graphpad software (n = 5, *: P<0.05, **: P<0.005, ***: P<0.0005). Representative of 3 independent experiments.

In vaccinated mice, we observed a strong IFN-γ response only after restimulation with LRV1+ *Lg* but not after LRV1- *Lg* restimulation (Fig 7), this observation led us to conclude that IFN-γ produced was mediated solely by LRV1 specific lymphocytes. In contrast, lymphocytes from vehicle-vaccinated mice showed mostly IFN-γ after LRV1- *Lg* re-stimulation. This indicated that these mice mainly developed a *Leishmania*-specific IFN-γ response (Fig 7). In addition, in LRVc vaccinated mice, *Leishmania* specific T cells are producing residual levels of IL-4 and IL-10 in comparison to non-vaccinated counterparts.

These results demonstrate that unprotected mice had a mixed Th1/Th2 response principally directed against *Leishmania* parasite as compared to a strongly Th1-biased response against the LRVc in vaccinated mice.

### CD3+ T cells confer significant protection against LRV1+ *Lg* infection

**Fig 8:**
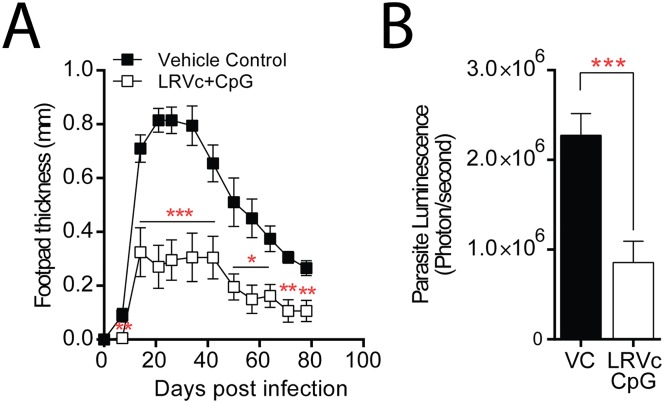
LRV1c Specific T cell transfer induced protection. C57BL/6 mice were vaccinated three times, 15 days apart, and sacrificed 7 days after the third vaccination. CD3+ T cells from vaccinated, or PBS injected mice (vehicle control), were purified (see Materials and Methods) and transferred into naïve C57BL/6 mice. These mice were infected 1 day after with LRV1+ *Lg* parasites. (A) Change in footpad swelling of mice infected with LRV1+ *Lg*. White squares represent the group carrying CD3+ cells from vaccinated mice, black squares indicate mice carrying vehicle control (B) Parasite load measured by *in vivo* luminescence at the peak of infection. Results are means±SEM. (A) Statistical significance tested by a two-way ANOVA, using Prism5 Graphpad software (n = 5, *: P<0.05, **: P<0.005, ***: P<0.0005). Representative of 3 independent experiments. (B) Statistical significance tested by a 2-tailed Student’s t-test using Prism5 Graphpad software (n = 5, *: P<0.05, **: P<0.005, ***: P<0.0005). Representative of 2 independent experiments.

In order to confirm the relevance of T cells in the protective mechanism against LRV, we performed cell transfer experiments. To this end, we isolated CD3+ T cells from the spleens of LRV1c+CpG immunized mice or their non-immunized controls and injected them intravenously (IV) into naïve C57BL/6 mice 2 days before the infection with LRV1+ *Lg*. As expected, mice re-injected with immunised cells had less parasites and smaller lesions than those receiving cells from non-immunised controls (Fig 8A and 8B).

In regard to previous results pointing out the major secretion of IFN-γ by LRV specific T cells, we repeated the cell transfer experiment using purified CD4+ or CD8+ t cells.

Results showing the importance of the synergy between this two subtypes of cells (S5 Fig). Furthermore, we performed the same experiment using CD3+ T cells purified from immunized IFN-γ KO mice. Results showed loss of protection in mice re-injected with IFN-γ KO CD3+ T cells (S6 Fig). These experiments allowed us to confirm that the immunological mechanism of LRV1c vaccination is driven by CD3+ T cells and their ability to secrete IFN-γ.

## Discussion

In this study, we focused on *L*. *guyanensis* parasites carrying a cytoplasmic virus, which acts as a virulence factor, exacerbating pathology in a murine model and responsible for treatment failure in human patients [4,5]. In previous studies it was shown that, the mechanism of LRV1 pathology occurs via its ability to act as a potent immunogen, inducing an anti-viral immune response. As immunogenicity is the cardinal requirement of a vaccine candidate, we tested the LRV1 viral capsid for its ability to confer protection as a prophylactic vaccine. We thus produced an *E*. *coli* recombinant protein (LRV1c) (S1 Fig). The antigenicity of LRV1c could be verified in immunized C57BL/6 mice that generated specific anti-capsid antibodies. We confirmed that the humoral response did not contribute to our vaccine-mediated immunity as serum from immunised mice failed to confer protection to their naïve counterparts. This humoral response was ineffective against LRV1, possibly because LRV1 resides as an endosymbiont inside *Leishmania* parasites and is likely not released in the extracellular environment as it would occur in most viral infections. Interestingly, by formulating LRV1c with CpG-ODN 1826, we were able to polarise the immunisation to a more appropriate Th1 cell-mediated response. This conferred a significant protective response against LRV1+ *Lg*, decreasing the lesion size and parasite burden in infected mice. Thus, our approach not only decreased lesional swelling, but also had a beneficial side-effect, caused by the high amount of IFN-γ secreted by LRVc-specific memory T cells, which impacted on parasite survival. Additionally receiving CD3+ T-cells from vaccinated mice were as protective as the LRV1c immunised group, which was not the case for mice receiving CD3+ T cells isolated from IFN-γ deficient mice. Thus, we identified a CD3+ T cell population, which reacted to LRV1c *ex vivo*, producing the signature Th1 cytokine IFN-γ. Within this cellular compartment, both CD4+ and CD8+ cells contributed to the IFN-γ production and we found that this synergy was essential to the immunisation mechanism, where neither CD4+ nor CD8+ cells were able to confer protection in naïve mice on their own. Thus, we also indirectly confirmed the fundamental pathological impact of LRV1 during *Leishmania* infection and showed that an immuno-prophylactic intervention could prevent LRV-driven pathology.

We hypothesise that the CpG ODN-1826 adjuvant is central to the mechanism of protection, where it likely created a potent Th1-based memory response, as has been previously described [31–33]. Importantly, however, the adjuvant had no effect on its own (S3 Fig) providing further indication that protection was offered via an LRV1c-specific response. We proposed that CD3+ memory T cells could react immediately after infection with LRV1+ *Lg* and polarized the immune response towards a protective Th1 phenotype already 16h post infection.

As anticipated with a cell-mediated response, we also observed a reduction in parasite load. This was possibly not only due to the direct targeting of cells presenting LRV1c antigens on MHC complexes, but also to the rapid and intense Th1 immune response within the lesion.

Taken together, we presented a novel vaccine strategy, whereby targeting persistent intracellular viruses within eukaryotic pathogens might reduce pathology induced by this hyperpathogen. This prophylactic approach could be important in specific endemic regions with a high prevalence of LRV1+ *Leishmania*, such as French Guyana, where more than 70% of the *Leishmania spp*. isolates carry LRV1 [34].

Such a vaccine approach could be useful for other pathogens carrying members of the *Totiviridae* family such as *T*. *vaginalis* and *G*. *lamblia*. It should also be mentioned that the yeast *Totivirus* capsid was engineered to express foreign antigens and could serve to aid future vaccine development [35]. Thus, the LRV viral capsid could possibly be used in a similar manner, expressing antigens from *Leishmania* parasites.

In conclusion, using a simple approach, we showed that it was possible to generate a protective immune response using capsid antigens from *Leishmania’*s viral endosymbiont, LRV1. The vaccine was able to diminish lesion exacerbation associated with LRV1 presence and in addition to reduce parasite burden via IFN-γ production as a beneficial side effect. This immunisation approach could be equally relevant as a therapeutic approach for patients infected with LRV1+ *Lg* or LRV1+ *Lb* at risk for disease relapse or failure of first-line therapy [4,5]. Furthermore, patients infected with LRV1+ *Leishmania* could first be screened for LRV1 presence and then provided with the vaccination to reduce metastatic risk, or perhaps reverse the complications of established metastasis as a therapeutic vaccine.

## Supporting Information

S1 Fig LRV1c recombinant protein. Recombinant LRV1c protein (1μg) was separated on a 10% polyacrylamide denaturing gel, and then stained with Coomassie brilliant blue (left), or transferred to a nitrocellulose membrane and incubated overnight at 4°C with the g018d53 anti-capsid polyclonal antibody and revealed by ECL (right).
(TIF)

S2 Fig Antibody response. Blood was analysed 8 weeks post infection in order to characterize the type of immune response present in vehicle control, or LRV1c immunized mice. Sera was analysed by ELISA in order to detect LRVc specific IgG1 or IgG2c antibodies. Results are means±SEM. Statistical significance tested by a 2-tailed Student’s t-test using Prism5 Graphpad software (n = 5, *: P<0.05, **: P<.005, ***: P<0.0005).
(TIF)

S3 Fig Adjuvant control. Mice were injected 3 times intramuscularly with 10μg of LRV1c + 50μg CpG (white), with PBS (black) or 50μg CpG (grey), 2 weeks apart. After the third vaccination, mice were infected in the hind of the footpad with 3x10^6^ LRV1+ *Lg* parasites. Footpad thickness was measured weekly (A) and parasite load (B) was measured by bioluminescence at 4 weeks post-infection. Results are means ±SEM. Statistical significance tested by a two-way ANOVA, using Prism5 Graphpad software (n = 5, *: P<0.05, **: P<0.005, ***: P<0.0005). Representative of 2 independent experiments.
(TIF)

S4 Fig Serum transfer control. Serum from LRV1c+CpG immunized or non-immunized mice, was re-injected in naïve C57BL/6 mice. To assess for correct transfer, mice were bled 7 days post infection and the presence of LRVc-specific AB by ELISA were assessed. Serial dilutions of the sera were performed. A non-saturated concentration was used for the figure. Black circle represents the five mice which received sera from VC mice. Oppositely, the white circle represents mice which received sera from LRVc + CpG immunized mice. Sera from vaccinated mice were used as a control (grey).
(TIF)

S5 Fig LRV1c Specific CD4+ or CD8+ T cell transfer are unable to confer protection. C57BL/6 mice were vaccinated three times 15 days apart and sacrificed 7 days after the third vaccination. CD4+ or CD8+ T cells from C57BL/6 vaccinated or PBS injected mice (vehicle control) were purified (see Materials and Method) and transferred into naïve C57BL/6 mice. These mice were infected 1 day after with LRV1+ *Lg* parasites. (A) Change in footpad swelling of mice infected with LRV1+ *Lg*. Black squares indicate mice carrying vehicle control CD3+ T cells, grey rhombus are mice receiving CD8+ T cells, and the light green circle are vaccinated mice receiving CD4+ T cells from vaccinated mice.(B) Parasite load measured by *in vivo* luminescence at the peak of infection. Results are means±SEM. (A) Statistical significance tested by a two-way ANOVA, using Prism5 Graphpad software (n = 5, *: P<0.05, **: P<0.005, ***: P<0.0005). Representative of 1 independent experiment. (B) Statistical significance tested by a 2-tailed Student’s t-test using Prism5 Graphpad software (n = 5, *: P<0.05, **: P<.005, ***: P<0.0005).
(TIF)

S6 Fig LRV1c Specific IFN-γ KO T cell transfer is unable to confer protection. C57BL/6 or IFN-γKO mice were vaccinated three times 15 days apart and sacrificed 7 days after the third vaccination. CD3+ T cells from C57BL/6, or IFN-γKO vaccinated, or PBS injected mice (vehicle control) were purified (see Materials and Method) and transferred into naïve C57BL/6 mice. These mice were infected 1 day after with LRV1+ *Lg* parasites. (A) Change in footpad swelling of mice infected with LRV1+ *Lg*. White squares represent the group carrying CD3+ cells from vaccinated C57BL/6 mice, black squares indicate mice carrying vehicle control and in grey CD3+ T cells from vaccinated IFN-γKO mice. (B) Parasite load measured by *in vivo* luminescence at the peak of infection. Results are means±SEM. (A) Statistical significance tested by a two-way ANOVA, using Prism5 Graphpad software (n = 5, *: P<0.05, **: P<0.005, ***: P<0.0005). Representative of 3 independent experiments. (B) Statistical significance tested by a 2-tailed Student’s t-test using Prism5 Graphpad software (n = 5, *: P<0.05, **: P<.005, ***: P<0.0005).
(TIF)
