# Supplementary figures and images for: Exacerbated Leishmaniasis Caused by a Viral Endosymbiont can be Prevented by Immunization with Its Viral Capsid
Source: PLoS Negl Trop Dis. 2017 Jan 18;11(1):e0005240. doi: 10.1371/journal.pntd.0005240 (PMC5242429; doi:10.1371/journal.pntd.0005240)

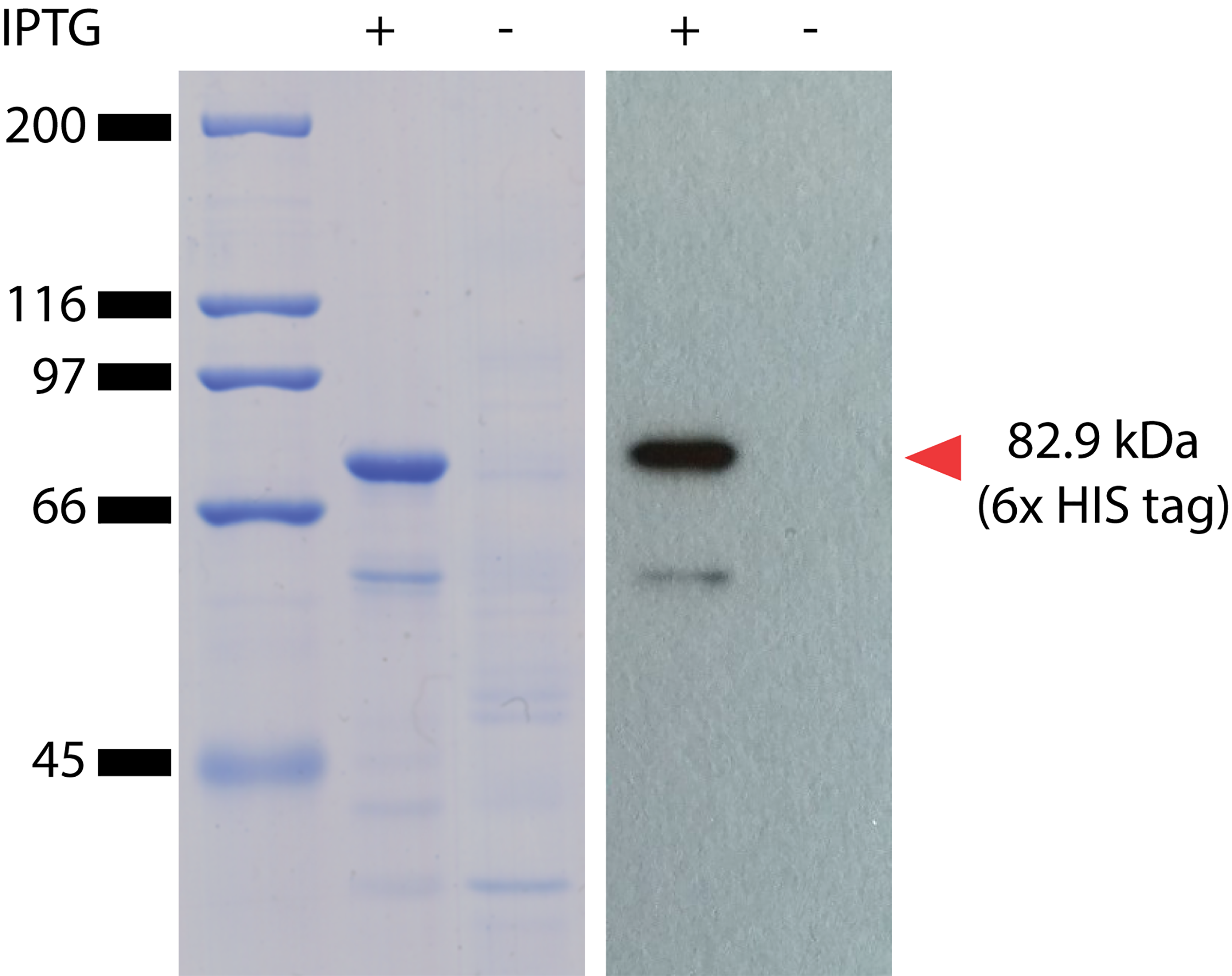

Supplement: S1 Fig — Recombinant LRV1c protein (1μg) was separated on a 10% polyacrylamide denaturing gel, and then stained with Coomassie brilliant blue (left), or transferred to a nitrocellulose membrane and incubated overnight at 4°C with the g018d53 anti-capsid polyclonal antibody and revealed by ECL (right). (TIF) [file pntd.0005240.s001.tif]

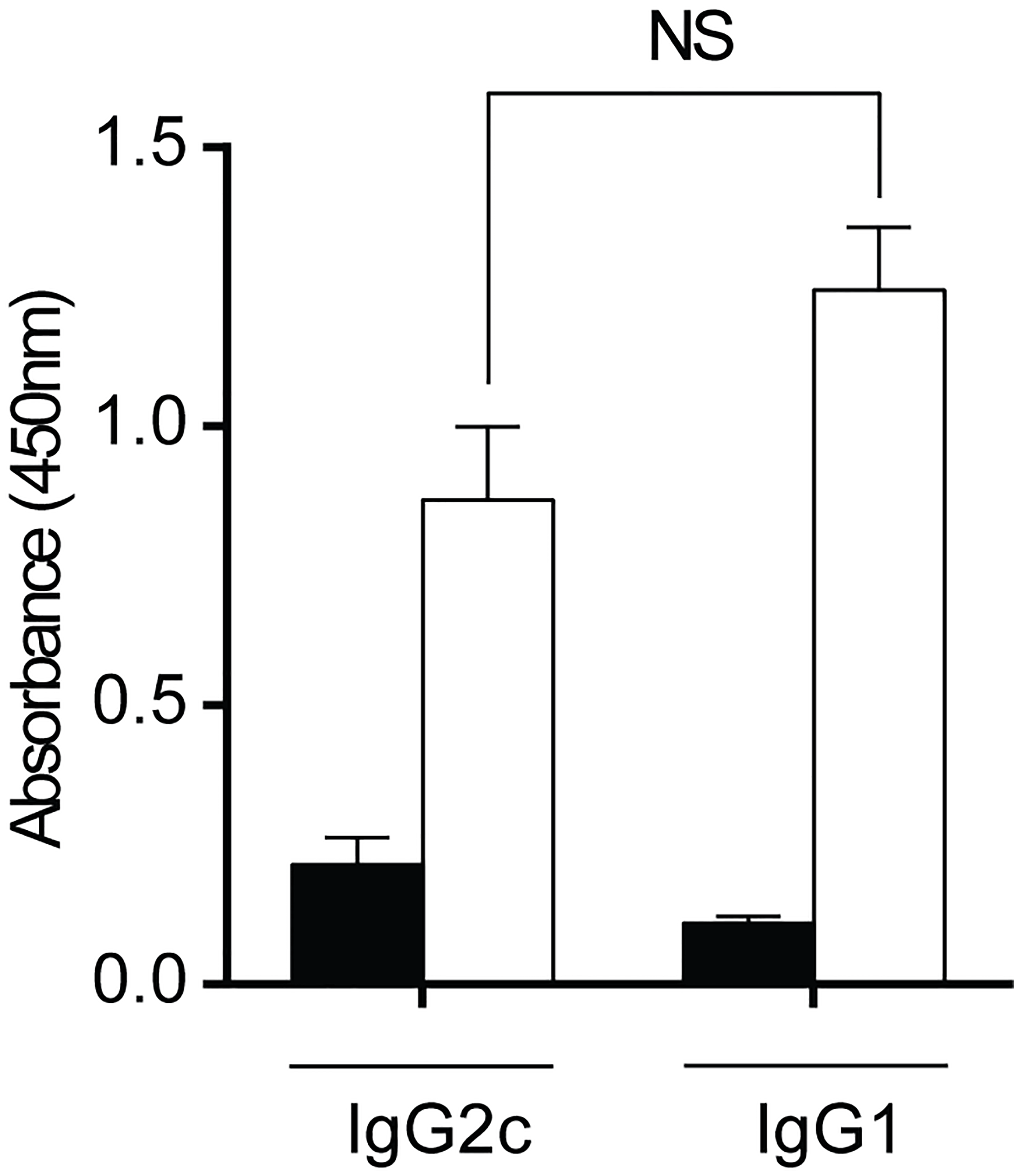

Supplement: S2 Fig — Blood was analysed 8 weeks post infection in order to characterize the type of immune response present in vehicle control, or LRV1c immunized mice. Sera was analysed by ELISA in order to detect LRVc specific IgG1 or IgG2c antibodies. Results are means±SEM. Statistical significance tested by a 2-tailed Student’s t-test using Prism5 Graphpad software (n = 5, *: P<0.05, **: P<.005, ***: P<0.0005). (TIF) [file pntd.0005240.s002.tif]

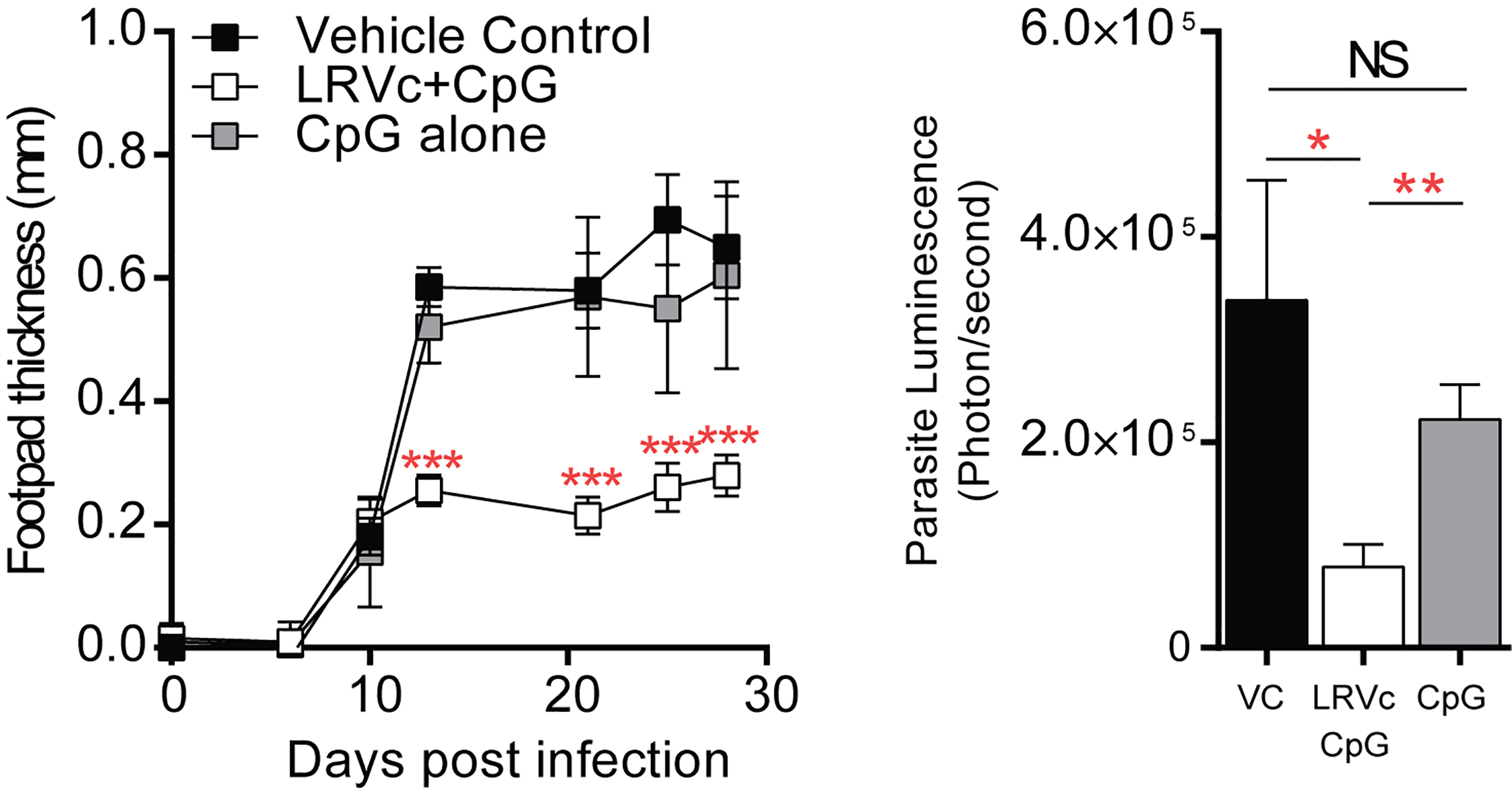

Supplement: S3 Fig — Mice were injected 3 times intramuscularly with 10μg of LRV1c + 50μg CpG (white), with PBS (black) or 50μg CpG (grey), 2 weeks apart. After the third vaccination, mice were infected in the hind of the footpad with 3x10^6^ LRV1+ Lg parasites. Footpad thickness was measured weekly (A) and parasite load (B) was measured by bioluminescence at 4 weeks post-infection. Results are means ±SEM. Statistical significance tested by a two-way ANOVA, using Prism5 Graphpad software (n = 5, *: P<0.05, **: P<0.005, ***: P<0.0005). Representative of 2 independent experiments. (TIF) [file pntd.0005240.s003.tif]

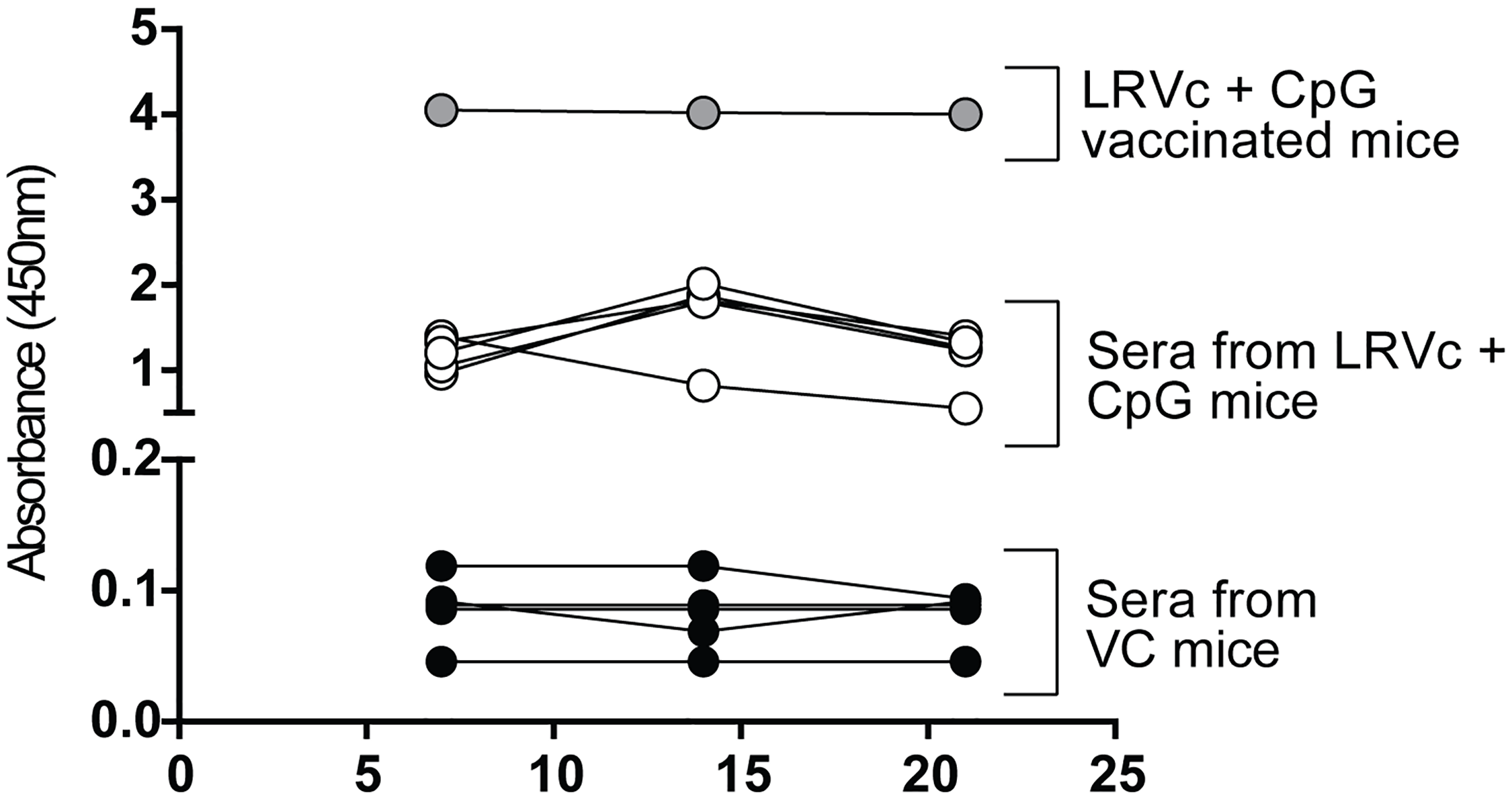

Supplement: S4 Fig — Serum from LRV1c+CpG immunized or non-immunized mice, was re-injected in naïve C57BL/6 mice. To assess for correct transfer, mice were bled 7 days post infection and the presence of LRVc-specific AB by ELISA were assessed. Serial dilutions of the sera were performed. A non-saturated concentration was used for the figure. Black circle represents the five mice which received sera from VC mice. Oppositely, the white circle represents mice which received sera from LRVc + CpG immunized mice. Sera from vaccinated mice were used as a control (grey). (TIF) [file pntd.0005240.s004.tif]

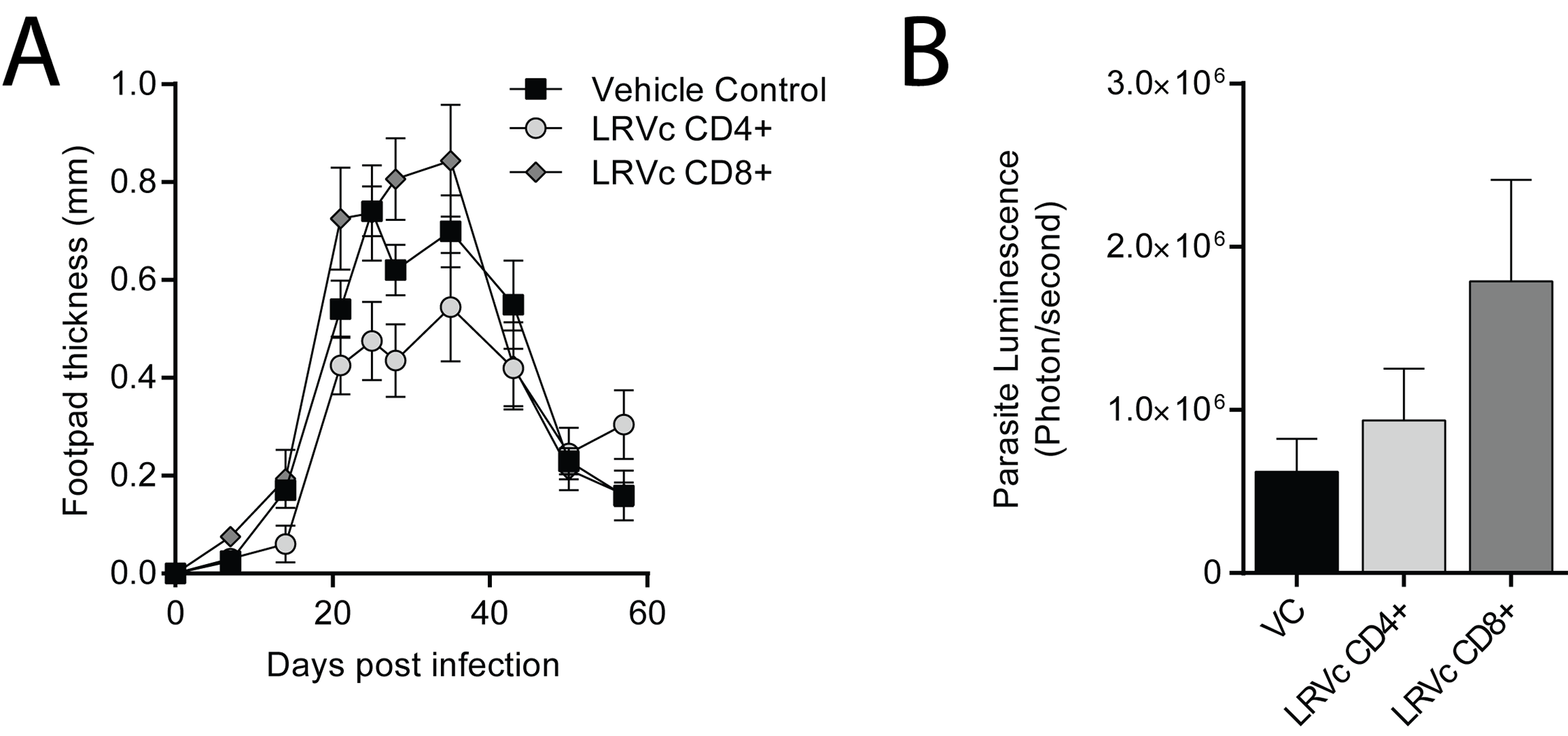

Supplement: S5 Fig — C57BL/6 mice were vaccinated three times 15 days apart and sacrificed 7 days after the third vaccination. CD4+ or CD8+ T cells from C57BL/6 vaccinated or PBS injected mice (vehicle control) were purified (see Materials and Method) and transferred into naïve C57BL/6 mice. These mice were infected 1 day after with LRV1+ Lg parasites. (A) Change in footpad swelling of mice infected with LRV1+ Lg. Black squares indicate mice carrying vehicle control CD3+ T cells, grey rhombus are mice receiving CD8+ T cells, and the light green circle are vaccinated mice receiving CD4+ T cells from vaccinated mice.(B) Parasite load measured by in vivo luminescence at the peak of infection. Results are means±SEM. (A) Statistical significance tested by a two-way ANOVA, using Prism5 Graphpad software (n = 5, *: P<0.05, **: P<0.005, ***: P<0.0005). Representative of 1 independent experiment. (B) Statistical significance tested by a 2-tailed Student’s t-test using Prism5 Graphpad software (n = 5, *: P<0.05, **: P<.005, ***: P<0.0005). (TIF) [file pntd.0005240.s005.tif]

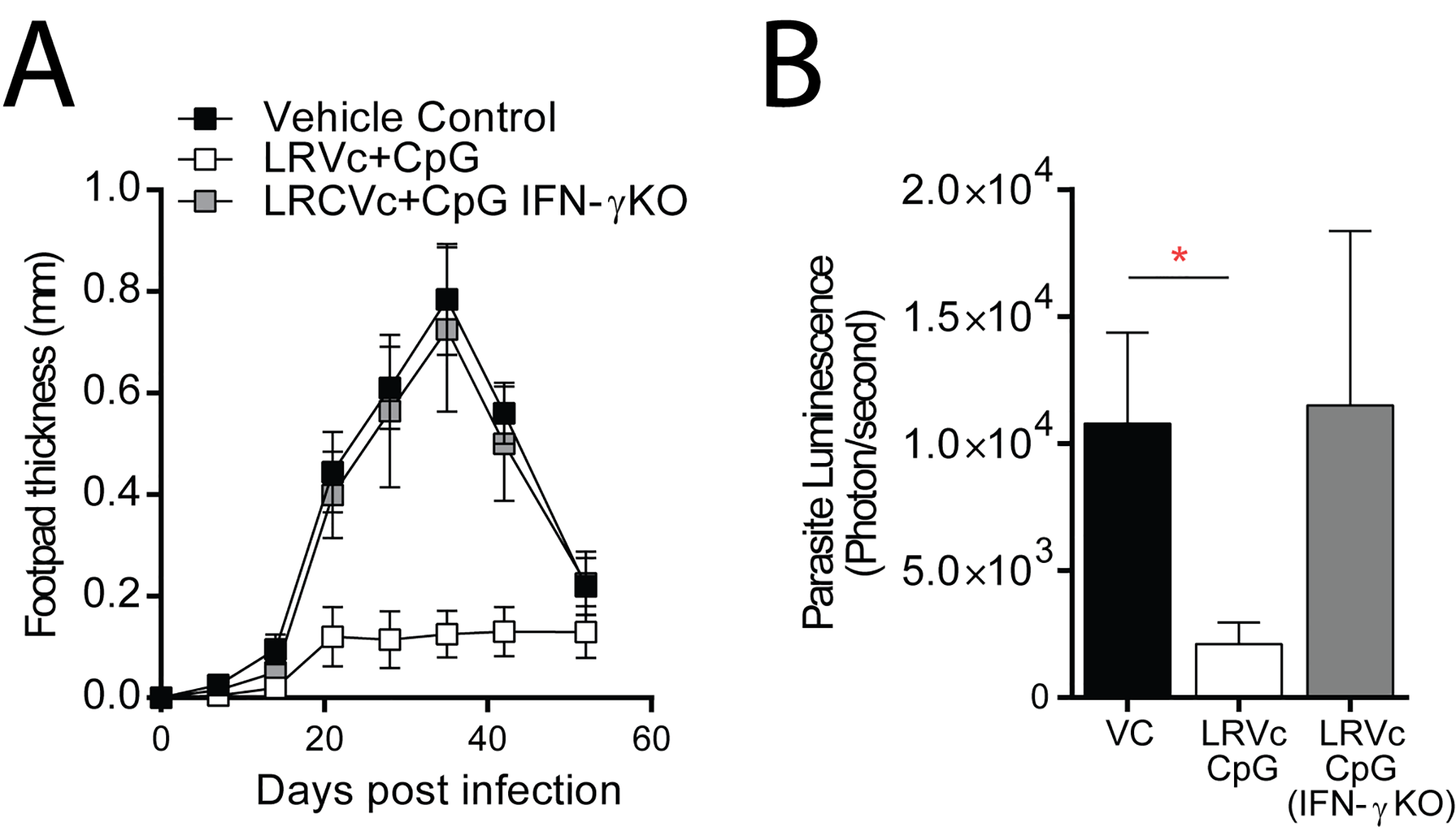

Supplement: S6 Fig — C57BL/6 or IFN-γKO mice were vaccinated three times 15 days apart and sacrificed 7 days after the third vaccination. CD3+ T cells from C57BL/6, or IFN-γKO vaccinated, or PBS injected mice (vehicle control) were purified (see Materials and Method) and transferred into naïve C57BL/6 mice. These mice were infected 1 day after with LRV1+ Lg parasites. (A) Change in footpad swelling of mice infected with LRV1+ Lg. White squares represent the group carrying CD3+ cells from vaccinated C57BL/6 mice, black squares indicate mice carrying vehicle control and in grey CD3+ T cells from vaccinated IFN-γKO mice. (B) Parasite load measured by in vivo luminescence at the peak of infection. Results are means±SEM. (A) Statistical significance tested by a two-way ANOVA, using Prism5 Graphpad software (n = 5, *: P<0.05, **: P<0.005, ***: P<0.0005). Representative of 3 independent experiments. (B) Statistical significance tested by a 2-tailed Student’s t-test using Prism5 Graphpad software (n = 5, *: P<0.05, **: P<.005, ***: P<0.0005). (TIF) [file pntd.0005240.s006.tif]
